# Species-Specific Conservation of Linear Antigenic Sites on Vaccinia Virus A27 Protein Homologs of Orthopoxviruses

**DOI:** 10.3390/v11060493

**Published:** 2019-05-29

**Authors:** Henrike P. Ahsendorf, Li L. Gan, Kamal H. Eltom, Ahmed Abd El Wahed, Sven-Kevin Hotop, Rachel L. Roper, Ulrike Beutling, Mark Broenstrup, Christiane Stahl-Hennig, Ludwig E. Hoelzle, Claus-Peter Czerny

**Affiliations:** 1 Division of Microbiology and Animal Hygiene, Department of Animal Sciences, University of Goettingen, Burckhardtweg 2, 37077 Goettingen, Germany; abdelwahed@gwdg.de; 2 German Primate Center, Leibniz-Institute for Primate Research, Unit of Infection Models, Kellnerweg 4, 37077 Goettingen, Germany; Li_lin.gan@web.de (L.L.G.); stahlh@dpz.eu (C.S.-H.); 3 Unit of Animal Health and Safety of Animal Products, Institute for Studies and Promotion of Animal Exports, University of Khartoum, 13314 Shambat, P.O. Box 32; 11115 Khartoum North, Sudan; keltom@gwdg.de; 4 Helmholtz Centre for Infection Research, Inhoffenstraße 7, 38124 Braunschweig, Germany; Sven-Kevin.Hotop@helmholtz-hzi.de (S.-K.H.); Ulrike.Beutling@helmholtz-hzi.de (U.B.); Mark.Broenstrup@helmholtz-hzi.de (M.B.); 5 Brody Medical School, East Carolina University, Greenville, NC 27834, USA; roperr@ecu.edu; 6 Institute for Animal Sciences, Livestock Infectiology and Environmental Hygiene, University of Hohenheim, Garbenstrasse 30, 70599 Stuttgart, Germany; ludwig.hoelzle@uni-hohenheim.de

**Keywords:** Vaccinia virus A27 protein homologs, epitope mapping, phylogenetic epitope variation, neutralizing antibodies

## Abstract

The vaccinia virus (VACV) A27 protein and its homologs, which are found in a large number of members of the genus *Orthopoxvirus* (OPXV), are targets of viral neutralization by host antibodies. We have mapped six binding sites (epitopes #1A: aa 32–39, #1B: aa 28–33, #1C: aa 26–31, #1D: 28–34, #4: aa 9–14, and #5: aa 68–71) of A27 specific monoclonal antibodies (mAbs) using peptide arrays. MAbs recognizing epitopes #1A–D and #4 neutralized VACV Elstree in a complement dependent way (50% plaque-reduction: 12.5–200 µg/mL). Fusion of VACV at low pH was blocked through inhibition of epitope #1A. To determine the sequence variability of the six antigenic sites, 391 sequences of A27 protein homologs available were compared. Epitopes #4 and #5 were conserved among most of the OPXVs, while the sequential epitope complex #1A–D was more variable and, therefore, responsible for species-specific epitope characteristics. The accurate and reliable mapping of defined epitopes on immuno-protective proteins such as the A27 of VACV enables phylogenetic studies and insights into OPXV evolution as well as to pave the way to the development of safer vaccines and chemical or biological antivirals.

## 1. Introduction

The genus *Orthopoxvirus* (OPXV) contains a group of large and closely related DNA viruses within the family Poxviridae, encompassing viruses that replicate in the cytoplasm of vertebrate or invertebrate cells [1,2]. Vaccinia virus (VACV), the prototype of the genus, was applied as the vaccine against the related Variola virus (VARV). This vaccination campaign led to the eradication of smallpox [3,4]. Immunization with VACV elicits potent B- and T-cell mediated immune responses, which provide cross protection against all the other OPXVs [5]. Currently, the majority of humans worldwide have no longer a protective immunity against poxviruses because of the termination of the vaccination campaign four decades ago. Therefore, there is considerable concern about the use of VARV and monkeypox virus (MPXV) as potential biological weapons [6,7], particularly after recent outbreaks of MPXV in the Democratic Republic of Congo, the United States of America, Nigeria and the United Kingdom [8,9,10] and as well as being reported by the World Health Organization [11]. Moreover, reservoirs for other closely related OPXVs, e.g., cowpox virus (CPXV), exist in the environment and may also endanger human health under certain circumstances [2,4,12,13,14,15], especially in immuno-compromised humans [16,17,18,19,20]. Therefore, it is crucial to join forces in the development of safer vaccines, antiviral agents, and protective human recombinant antibodies for passive immunization.

VACV contains a double-stranded DNA genome of approximately 194,000 nt, depending on the strain, encoding more than 200 polypeptides [21]. Morphogenesis results in two distinct infectious forms of virus particles [22,23]. The majority consists of the fully functioning intracellular mature virus (IMV) with a single envelope, as well as a small proportion of extracellular enveloped virus (EEV), which is surrounded by an additional Golgi-derived envelope. IMV is the predominant infectious form remaining within the infected cell and mediating host-to-host transmission, whereas EEVs, on the other hand, are important for direct cell-to-cell transmission inside the host [24,25,26,27,28,29,30,31]. Viral particles linked to the outer surface of the cell have been visualized by electron microscopy and were named cell-associated enveloped virus (CEV) [22,24].

Vaccination results in the induction of neutralizing antibodies against several VACV envelope proteins. Structural proteins of immunological relevance containing targets for neutralizing antibodies were identified on both IMV (including A27, D8, H3, A17, and L1), and EEV/CEV (including A33 and B5) [15,32,33,34,35,36,37,38]. Most importantly, these proteins led to the induction of protective immunity in vivo [32,35,38,39,40,41,42,43,44,45,46,47,48,49,50,51]. One of the best characterized and intensively studied IMV envelope proteins is the A27 protein [32,52,53,54,55], encoded by a gene corresponding to the VACV Copenhagen open reading frame (ORF) A27L [42,56]. This protein is present in all members of OPXVs, forms a trimeric structure on the surface of IMVs, and binds to the glycosaminoglycan (GAG) heparan sulfate on the surface of mammalian cells [13,14] by a turn-like structure, which is formed by a KKPE segment [57]. Additionally, the A27 protein builds a complex together with four other membrane proteins (A14, A17, A25 and A26). Because A27 lacks its own trans-membrane domain, its association with A17 mediates the anchorage within the envelope of IMVs [14,58,59,60]. The 110 amino acids of the A27 protein can be divided into four functional areas: an N-terminal signal peptide, a Lys/Arg-rich heparin binding domain (HBD), an α-helical coiled-coil domain (CCD), and a C-terminal leucine zipper motif (LZD) [13,59,61]. The HBD (aa 21–34) including the KKPE segment (aa 26–29), is essential for binding to heparan sulfate [34,57,61,62]. The CCD (aa 43–84) possesses the two cysteine residues 71 and 72, which are responsible for forming disulfide bonds with the A26 protein [61,63]. The LZD (aa 85–110) is considered to be the binding region of A17 [13,61,64,65]. A27 is important for virus replication, as it regulates cell entry and virus egress. Conditional lethal mutant independent assays like isopropyl-o-thiogalactoside (IPTG)-induced expression of the A27 protein during infection restores the interaction of IMV with Golgi-derived membranes leading to EEV formation. Thus, the A27 protein is essential for the envelopment of IMV by Golgi membrane and for their subsequent egress from the cell [66]. The A27 protein was designated as the fusion protein, because monoclonal antibodies binding to this protein of 14 kDa were able to block fusion [52,54]. However, more recent evidence suggests it is more likely that a complex of at least 11 envelope proteins is responsible for fusion [36,37]. The A27 protein, however, is not integrated within this complex.

Here, we have identified six linear epitopes recognized by A27 mAbs [40] using SPOT synthesis on cellulose membranes and peptide microarray technology. Affinities were investigated and neutralization capabilities of the mAbs were improved after the addition of human complement. The identified epitopes toward the far ends of A27 were conserved among OPXV upon screening all A27 sequences available in the GenBank, while the centrally located epitopes were species-specific. 

## 2. Materials and Methods

### 2.1. Cells and Viruses

The permanent monkey kidney cell line MA-104 cultured in minimum essential medium (MEM) (PAN-BIOTECH, Aidenbach, Germany) and supplemented with 7% fetal calf serum (FCS), was used to propagate the VACV strains Bern, CVA, Elstree, IHD-J, Copenhagen wild type (WT), Copenhagen host range (HR), R325, TT, the neuro-vaccinia virus strains Hagen, Levaditi and Munich 1, as well as the OPXV strains camelpox virus (CMLV) CP1, CPXV KR2 Brighton, mousepox virus (ectromelia; ECTV) Munich 1, and MPXV Copenhagen (for references see [39]). For virus propagation FCS was reduced to 2%. Infectivity titers were determined on 24-well plates (Nunc, Wiesbaden, Germany) and calculated as plaque forming units (pfu/mL). For plaque reduction tests, Vero cells cultured in MEM, supplemented with 5% FCS were used and maintained in the same way as MA-104. For syncytium formation and fusion experiments, BS-C-1 cells cultured in MEM, supplemented with 10% FCS were used to propagate the VACV strain Western Reserve (WR). Virus multiplication was carried out in MEM with 2.5% FCS as described before [31,67].

The Modified VACV Ankara (MVA) was grown in primary embryonic chicken fibroblast cells (CEF). Due to its micro-plaque generation, infectivity titer was calculated as TCID_50_/mL after titration in 96-well microplates. The culture medium was MEM containing 2.5% FCS. 

All virus preparations were purified and concentrated by sucrose gradient centrifugation as previously described [40,68]. The purified preparations consisted of intracellular mature virus (IMV). Protein contents of the samples were determined according to the method of Lowry et al. [69].

### 2.2. Polyclonal and Monoclonal Antibodies

Polyclonal rabbit hyperimmune sera and monoclonal BALB/c-mouse antibodies against purified VACV MVA, VACV Munich 1, CPXV KR2 Brighton, ECTV Munich 1, and MPXV Copenhagen were prepared as described elsewhere [39,40]. The monoclonal antibodies (mAbs) were cross-reactive against other OPXVs in a species-specific manner. For this study, the cross-reactive but A27-specific mAbs anti-VACV 5B4/F2 (epitope #1A), anti-VACV 2C11/1B4 (epitope #1B), anti-CPXV 3F5/2D5 (epitope #1C), anti-CPXV 1D5/1E10 (epitope #1D), anti-ECTV 2G8/1E4 (epitope #4), and anti-ECTV 5B1/2G6 (epitope #5) were used. Monoclonal antibodies from cell culture supernatants or polyclonal hyper-immune sera were purified on Protein G sepharose columns (HiTrap™ 5 mL Protein G HP, Sigma Aldrich, Taufkirchen, Germany), dialyzed against phosphate-buffered saline (PBS) and sterilized by centrifugation at 20,238× *g*. Protein contents of the antibody preparations were determined according to the method of Lowry et al. [69].

### 2.3. Plaque Reduction Test

The neutralization potency of six A27-specific mAbs was tested by plaque reduction test (PRT) against VACV Elstree as reference strain. Purified antibodies were diluted with MEM (PAN-BIOTECH, Aidenbach, Germany) and adjusted to a concentration of 400 µg/mL. A volume of 125 µL of the antibody preparations was titrated in two-fold serial dilutions on 96-well microplates containing 125 µL/well MEM supplemented with 2.5% FCS to avoid antibody coating. After antibody titration, one dilution series received 1% sterile human complement (Sigma Aldrich, Taufkirchen, Germany) per well, the other remained free of complement. Then, 100 pfu (125 µL) of VACV Elstree was added to each well. As plaque-forming control, 250 µL MEM/well with or without 0.5% human complement, containing 100 pfu VACV Elstree was used. The virus negative control was 250 µL MEM/well alone with or without 0.5% human complement. After incubation of the 96-well microplates at 37 °C for one hour, the mixtures were transferred to 24-well plates containing a confluent monolayer of Vero cells. After incubation at 37°C for one hour, the supernatants were poured out and replaced by 0.5 mL MEM containing 2.5% FCS and 0.5% methyl cellulose (Sigma Aldrich, Taufkirchen, Germany). The plates were then incubated at 37 °C for 48 h, before the cells being fixed and stained with a solution containing 25% formaldehyde, 8.5% ethanol and 1.5% crystal violet. The plaques were counted by visual inspection while illuminated. Neutralization was determined as ≥50% plaque reduction compared to the virus control. Each PRT was performed in triplicates 

### 2.4. Inhibition of Cell Fusion and Syncytium Formation

Cell fusion experiments were performed as described before [31,67,70]. Confluent BS-C-1 monolayers cultured in MEM with 2.5% FCS in 24-well plates (1 mL/well) were infected with 100 pfu/well VACV WR for 1 h at 37 °C, washed twice and incubated either with warm medium alone or with warm MEM containing purified mAbs (200 µg/mL). Then, 24 h post infection, the cells were incubated for 3 min at 37 °C at pH 4.8 with warm fusion buffer (phosphate-buffered saline with 10 mM 2-(*N*-morpholino)ethanesulfonic acid and 10 mM HEPES). The cells were washed twice with warm MEM (treated for two min at 37 °C). Afterwards, warm medium (MEM + 1% FCS), with or without mAbs (200 µg/mL), was added again. The cells were incubated for 4 h at 37 °C and then observed by phase-contrast microscopy. An indicator for cell fusion was the formation of syncytia, which are large, structure-less, fused cell areas [54].

### 2.5. Binding Affinities of the Monoclonal Antibodies (mAbs) in Indirect Enzyme-Linked Immunosorbent Assays (ELISAs)

For quantification of the binding affinities of mAbs to different OPXVs, an indirect enzyme-linked immunosorbent assay (ELISA) was applied; 96-well microplates were coated with 1 µg/mL of the VACV strains Bern, CVA, Elstree, IHD-J, Copenhagen wild type (WT), Copenhagen host range (HR), R325, TT, the neuro-vaccinia virus strains Hagen, Levaditi and Munich1, the modified VACV Ankara (MVA) as well as the OPXV strains camelpox virus (CMLV) CP1, cowpox virus (CPXV) KR2 Brighton, mousepox virus (ectromelia; ECTV) Munich 1, and MPXV Copenhagen in carbonate/bicarbonate buffer (pH 9.6; 100 µL/well). After blocking with 2% skimmed milk and 10% fetal calf serum in PBS, purified mAbs adjusted to a concentration of 50 µg/mL were titrated in two-fold serial dilutions (100 µL/well). Incubation was performed at 37 °C for 1 h. After five washing steps with PBS, peroxidase conjugated goat anti-mouse IgG (whole molecule; Sigma Aldrich, Taufkirchen, Germany) produced in goats was added to the 96-well microplate in a working dilution of 1:2000 (100 µL/well) and incubated at 37 °C for 1 h. Thereafter, the plate was washed five times with PBS again, before the developing solution (3, 3′, 5, 5′ tetramethylbenzidine; Abcam, Cambridge, UK) was added (100 µL/well). The reaction was stopped by 1 N hydrochloric acid (50 µL/well). The OD-values were measured by a photometric plate reader (TECAN Sunrise plate reader with the Magellan complete software, Männedorf, Switzerland) at a wavelength of 450 nm. Affinity was calculated from the average absorption of the triplicates using Michaelis-Menten kinetics [71,72] and the program GraphPad Prism version 7.00 for Mac (La Jolla, CA, USA).

### 2.6. Epitope Mapping by SPOT Synthesis on Cellulose Membranes

The whole A27 protein of VACV Copenhagen [52,56] representing 110 amino acids [56], was directly synthesized stepwise on derivatized cellulose membranes through 101 decapeptides with an offset of one aa (9 aa overlap). The synthesis on derivatized cellulose membranes using Fmoc-protected amino acid pentafluorophenyl or /V-hyroxyoxo-dihydro-benzotriazine esters and the screening were performed according to the method described before [73] and the manufacturer of the SPOTs kit (Cambridge-Research Biochemicals, ICI, representative in Germany IC-Chemikalien, Carl-Zeiss-Ring 15, Ismaning).

The reactivity of the generated peptides with mAbs was tested using β-galactosidase-labeled goat anti-mouse immunoglobulins (Abcam, Cambridge, UK) as secondary antibodies. The color development of the peptide spots occurred after treatment with 5-bromo-4-chloro-3-indolyl-β-d-galactopyranoside, the substrate for the β-galactosidase-labeled secondary antibodies.

### 2.7. Epitope Mapping by Microarray Scanning Chips

An OPXV microarray chip was designed as depicted in Figure S1. 15-mer peptides overlapping by 12 amino acids (3 aa offset) were synthesized via SPOT synthesis on a cellulose membrane [73], passed through the SC^2^ process [74] and spotted onto microscope glass slides. The chip contained eight identical arrays of 521 peptides each (Figure S1A). A total of 475 of those overlapping peptides represented the entire amino acid sequences of A27, D8, H3, L1, A33, and B5 proteins of VACV Western Reserve (Figure S1B, GenBank accession number: AY243312.1). Forty-six peptides were amino acid variations of VACV A27 and D8 proteins to the corresponding homologs of other OPXVs (Table S1). In addition, 10 cellulose-conjugated biotin spots served as a positive control and orientation for the SPOT Calling Program. The OPXV microarray chip was designed to screen four samples simultaneously. Therefore, each peptide was printed eight times to obtain technical replicates, which could be divided into four identical sub-arrays using an adhesive chamber (SecureSeal, Sigma-Aldrich Co. LLC, USA). In order to obtain equal antibody concentrations of 2 µg/µL per chamber, protein concentrations were measured using a NanoDrop ND-1000 Spectrophotometer. The screening procedure with the microarray chip was performed as previously reported [75].

### 2.8. DataBase Analysis of A27 Protein Sequences and Orthopoxvirus (OPXV) Phylogeny

All A27 protein sequences of different OPXV strains available until 31st August 2018 were downloaded from the NCBI GenBank database [76,77,77]. So, a total of 391 complete and partial A27 sequences were aligned by the Clustal W [78] option in the Lasergene MegAlign 12 software (DNAStar, Madison, WI, USA). The used data included sequences from Old World species such as VARV, VACV, buffalopox virus (BPXV), rabbitpox virus (RPXV), horsepox virus (HSPV), MPXV, CPXV, CMLV, ECTV, and taterapox virus (TaPXV) as well as the New World species raccoonpox virus (RCNV), volepox virus (VPXV), and skunkpox virus (SkPXV). A phylogenic tree was created with Geneious software (version 9.1.6, Biomatters Inc., Aukland, New Zealand), using not more than five sequences per epitope variant.

## 3. Results

### 3.1. Fine Mapping of the Vaccinia Virus (VACV) A27 Epitopes by SPOTs Membrane

**Figure 1:**
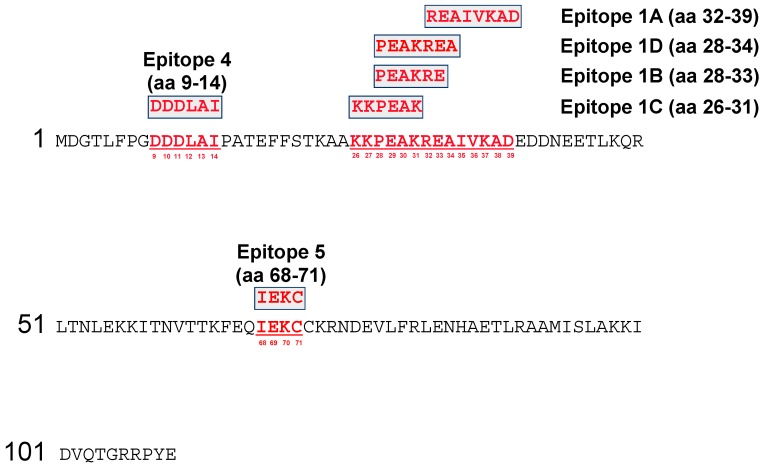
Mapping of the antigenic sites of the six A27-specific mAbs on a SPOTs membrane.

The targets of six anti-A27 mAbs were mapped by SPOT synthesis (Table S1). The A27 protein of VACV Copenhagen was synthesized on a SPOTs membrane in form of 101 decapeptides with 9 aa overlap to cover the whole sequence of 110 aa. Immunodetection was carried out with the six OPXV-specific mAbs (Figure 1). A positive reaction was indicated by blue coloration of those spots binding the corresponding antibody. The complex of the four closely related antigenic sites #1A–D was identified and located within the range of aa 26–39. Epitope #1A (mAb: 5B4/2F2) was directed against the sequence region of eight aa 32-REAIVKAD-39 (Figure S2). In case of the mAb 5B4/2F2, seven spots were recognized (No. 10–16), from which spots 11–13 showed the strongest reactivity to the mAb, which indicated an optimal antibody binding condition and only these peptides were used for defining the epitope. By the same procedure, epitope #1B (mAb 2C11/1B4) was assigned to the six aa 28-PEAKRE-33, epitope #1C (mAb 3F5/2D5) to the six aa 26-KKPEAK-31, and epitope #1D (mAb 1D5/2D11) to seven aa 28-PEAKREA-34 (Figure 1 and Table S2). Epitope #4 (mAb 2G8/1E4) was located at aa positions 9-DDDLAI-14, whereas epitope #5 (mAb 5B1/1A11) was represented by the four aa 68-IEKC-71. 

### 3.2. Fine Mapping of the VACV A27 Epitopes by Microarray Analysis

Similar mapping results were obtained when using the OPXV microarray chip imprinted with 521 pentdecapeptides with 12 aa overlap. Epitope #1A (mAb 5B4/2F2) was only one aa longer compared to the SPOTs membrane and was, therefore, directed to the sequence region aa 31-KREAIVKAD-39. Epitopes #1B (mAb 2C11/1B4), #1C (mAb 3F5/2D5) and #1D (mAb 1D5/2D11) were all assigned to the aa region 28-PEAKRE-33. For epitope #1B, the microarray chip and the SPOTs membrane yielded identical results. The epitope #1D was mapped to the same region, but only one aa shorter on the microarray chip. Epitope #4 (mAb 2G8/1E4) was allocated to aa 7-PGDDDLAIPATE-18 and, therefore, by 6 aa longer compared to results from the SPOTs membrane. MAb 5B1/1A11 (epitope #5), however, did not react with any of the peptides on the chip, although the target sequences detected on the SPOTs membranes were present in the microarray spots no. 20–23 and 493–496 (Tables S1 and S2 and Figure S3). In the following investigations, we refer, therefore, to the epitope locations provided by the SPOTs membrane, because they were regarded to be more accurate due to the shorter aa offset of one aa compared to three aa in the microarrays.

### 3.3. Identification of Neutralization-Mediating Epitopes with/without Complement

**Figure 2:**
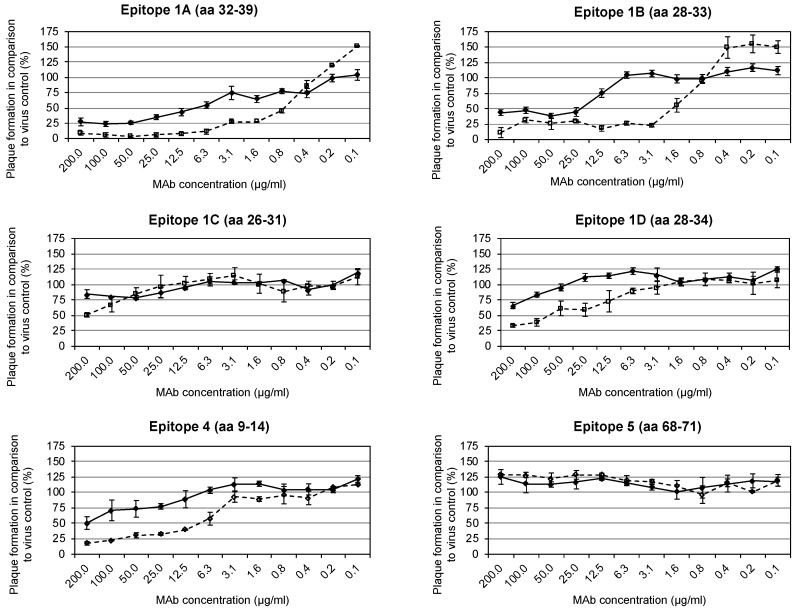
Neutralization-mediating epitopes were detected by plaque reduction test (PRT). Six mapped anti-A27 mAbs were incubated with VACV Elstree either in the presence or absence of 1% human complement. The plaque formation in comparison to virus control in percent is shown as a function of the respective concentration in µg/mL of the mAbs. The solid line (♦) shows the antibody alone, the dashed line (∎) the antibody together with 1% complement. The mAbs 5B4/2F2, 2C11/1B4, and 2G8/1E4 neutralized VACV Elstree in the absence of complement. Neutralization could be improved in the presence of complement. The mAbs 3F5/2D5 and 1D5/1E10 neutralized the VACV Elstree only in the presence of 1% complement, while no neutralization was observed with the mAb 5B1/2G6.

Epitopes able to induce neutralizing antibodies were detected by PRT. Protein G purified mAbs against the six antigenic sites mapped on the A27 protein were incubated with VACV Elstree either in the presence or absence of 1% human complement. Complement was used to increase the footprints of the mAbs on the viral surface. The mAbs 5B4/2F2 (epitope #1A), 2C11/1B4 (epitope #1B), and 2G8/1E4 (epitope #4) neutralized VACV Elstree (measured as 50% plaque reduction) in the absence of complement at concentrations of 12.5, 25 and 200 µg/mL, respectively (Table 1 and Figure 2). An 8- to 16-fold increase in the neutralization strength of these mAbs was observed in the presence of complement (5B4/2F2: 1.6 µg/mL; 2C11/1B4: 3.1 µg/mL; 2G8/1E4: 12.5 µg/mL). The mAbs 3F5/2D5 (epitope #1C) and 1D5/1E10 (epitope #1D) neutralized VACV Elstree only in the presence of 1% complement at concentrations of 200 µg/mL and 100 µg/mL, respectively, while no neutralization was observed with the mAb 5B1/2G6 (epitope #5).

### 3.4. Inhibition of Cell Fusion

**Figure 3:**
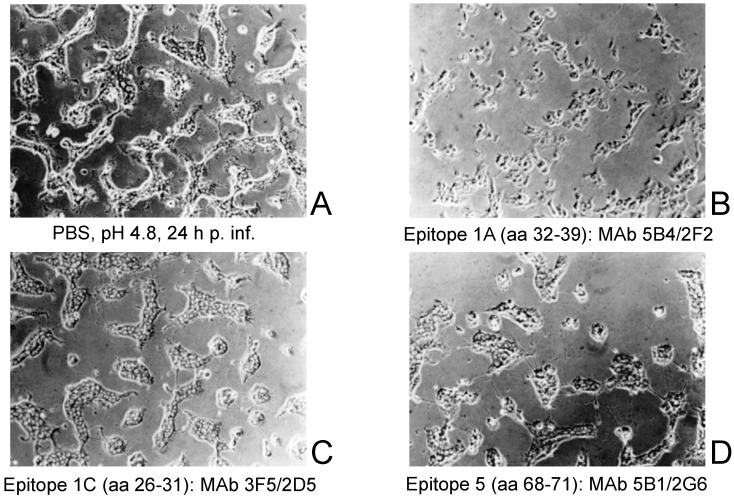
Acid-induced fusion inhibition experiments with VACV WR and three of the six anti-A27 mAbs. (**A**): Fusion of infected BS-C-1 cells was indicated by the formation of larger, structureless, and fused cell areas. (**B**): Fusion was inhibited by the mAb 5B4/2F2 directed to epitope #1A (aa 32–39). (**C**): The mAb 3F5/2D5 against epitope #1C (aa 26–31) was binding upstream of the mAb 5B4/2F2 and not able to block cell fusion. (**D**): The mAb 5B1/2G6 binding to the C-terminal epitope #5 (aa 68–71) was also not able to inhibit fusion.

A27 was initially designated as the fusion protein [54,79,80]. However, more recent evidence indicates that there is not only one fusion protein in the envelope of IMV, but rather a fusion complex consisting of at least 11 proteins [36,37,81]. Evidence now suggests that the A27 protein is not integrated into the fusion complex [36,82]. Other investigations reported a second fusion complex consisting of A17 and A27 [65], where the fusion event of VACV WR at pH 4.8 was inhibited by anti-A27 mAbs. Therefore, we retested this effect using three epitope-mapped anti-A27 mAbs from our collection to cover the entire target region. Fusion of infected BS-C-1 cells was indicated by the formation of large areas of fused cells, rather than separate individual cells (Figure 3A). Fusion was inhibited by the mAb 5B4/2F2 directed to epitope #1A (aa 32–39) (Figure 3B). The mAb 3F5/2D5 against epitope #1C (aa 26–31) was binding upstream of the mAb 5B4/2F2 and was not able to block cell fusion (Figure 3C). The same was observed for mAb 5B1/2G6 binding to the C-terminal epitope #5 (aa 68–71) (Figure 3D). 

### 3.5. Binding Affinities of the mAbs to Various Variants of OPXVs

**Figure 4:**
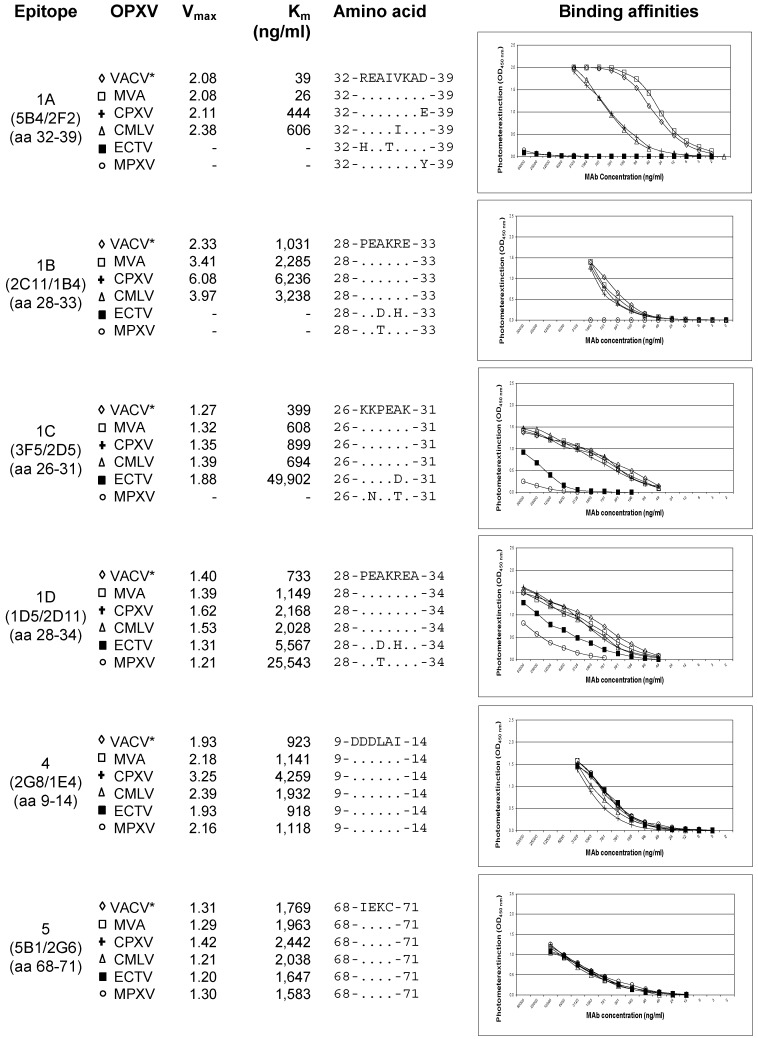
Binding affinities of the six anti-A27 mAbs determined by indirect ELISAs towards the reference strains VACV MVA, a mean of 11 different VACV strains, cowpox virus (CPXV) KR2 Brighton, camelpox virus (CMLV) CP1, mousepox virus (ECTV) Munich 1, and monkeypox virus (MPXV) Copenhagen. In case of the VACV strains, with the exception of VACV MVA, all data were calculated as mean values from the individual binding curves. The optical density (OD_450 nm_) is shown as a function of the respective concentration (ng/mL) of the mAb. Moreover, the aa sequence matches and differences of the epitopes, as well as the V_max_ and K_m_ values are shown. All mAbs directed to epitope complex #1A–D showed strong binding activity to VACV, CPXV and CMLV, but did not react or bound weakly to ECTV and MPXV. In contrast the mAbs targeting epitopes #4 and #5 showed the same strong binding activities to all OPXVs tested. All the six antigenic sites were recognized equally well in VACV MVA and in all the other VACVs. ♢ = Mean of 11 different VACV strains; ☐ = VACV MVA; **+** = CPXV KR2 Brighton; △ = CMLV CP1; ◼︎ = ECTV Munich 1; ￮ = MPXV Copenhagen.

Binding affinities of the purified mAbs to the six A27 epitopes detected in VACV Elstree were determined by indirect ELISAs on microplates coated with the purified reference strains VACV-MVA, VACV, CPXV KR2 Brighton, CMLV CP1, ECTV Munich 1, and MPXV Copenhagen. The binding curves were determined in triplicates for each virus strain. In case of the VACV strains, with the exception of VACV MVA, all data were calculated as mean values. VACV MVA was presented alone in order to compare affinity data directly to other VACV strains (Figure 4). All mAbs directed to epitope complex #1 showed strong binding activity to VACV, CPXV and CMLV, but did not react with or bound only weakly to ECTV and MPXV. In all VACV strains, the mAb 5B4/2F2 bound to its epitope #1A equally well. There was no difference in the amino acid sequence of the respective epitope. An 11.5-23-fold decrease in binding activity was observed with CPXV KR2 Brighton and CMLV CP1. Responsible for this finding were obviously the aa exchanges D39E in CPXV and V36I in CMLV. In ECTV Munich 1 and MPXV Copenhagen, the epitope #1A could not be detected, apparently due to aa exchanges R32H and I35T in ECTV and D39Y in MPXV. Epitope #1B was detected by the mAb 2C11/1B4 in VACVs, CPXV and CMLV with a similar affinity, whereas aa exchanges A30D and R32H in ECTV and A30T in MPXV caused the loss of the mAb reaction. Epitope #1C was also detected equally well in VACVs, CPXV and CMLV by the corresponding mAb 3F5/2D5. In ECTV, the kinetics of the mAb were reduced 25- to 53-fold according to the aa exchange A30D. In MPXV, the epitope was only very weakly detectable. The mAb 1D5/2D11 against epitope #1D, which is only one aa longer than epitope #1B (A at position 34), reacted equally well with VACVs, CPXV and CMLV. Despite the aa exchanges A30D and R32H in ECTV, which were also present, the mAb detected the epitope with 2.6 to 7.6-fold weaker affinity compared to VACVs, CPXV and CMLV. Even in MPXV, the epitope #1D was detected by the mAb, albeit with a 4.6- (ECTV) to 34.8-fold (VACVs) weaker intensity. In contrast to the heterogeneous species-specific binding behavior of mAbs directed to the epitope complex #1A–D, the mAbs targeting epitopes #4 and #5 showed the same strong binding activities to all OPXVs tested. V_max_- and K_m_-values were in the same range. 

### 3.6. Species-Specific Epitope Conservation and Variation among the OPXV Members

A total of 391 amino acid sequences of the OPXV A27 protein homologs from the GenBank were analyzed with respect to species-specific conservation or variation of the six sequential antigenic sites mapped.

Epitope #4, located at the N-terminus of the A27 protein between aa residues 9–14 (9-DDDLAI-14), is highly conserved within the genus OPXV (Tables S3–S5). This motif was found in 372 of the 391 analyzed sequences. 

The motif 68-IEKC-71 of epitope #5 is nearly genus-specific in OPXVs (Tables S4–S6). It was present in almost all OPXV strains (389/391). In case of 3/3 SkPXVs, the motif 68-IEKC-71 is postponed backward (94-IEKC-97) due to an insertion of 10 aa in the epitope complex #1A–D (as stated below) and 16 aa between epitope complex #1A–D and #5. The same displaced epitope region (94-IEKC-97) was observed in the case of VACV WR 65-16 (P26312.1) as well, because the A27 of this virus strain starts at aa 73L. 

**Figure 5:**
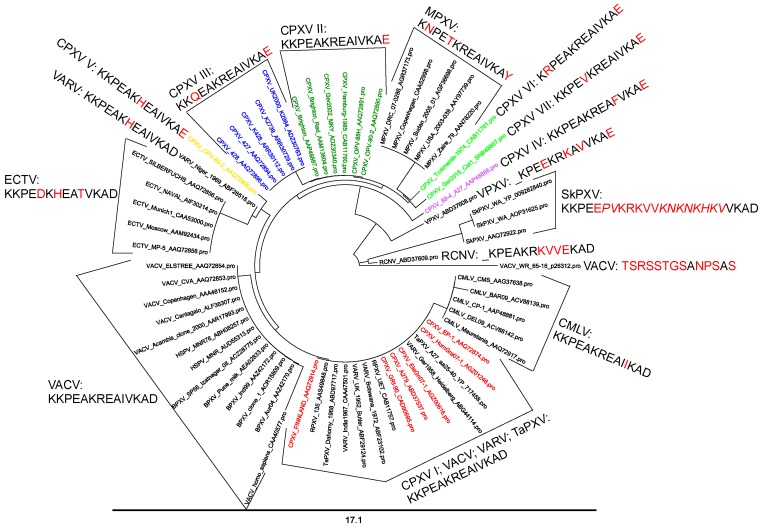
Phylogenetic analysis is based on the different OPXV amino acid sequences of the epitope complex #1A–D. The sequences were divided into two distinct groups: the monophyletic sequences, including VACV, VARV, ECTV, MPXV, CMLV, HSPV, RPXV, TaPXV, SkPXV, RCNV and VPXV are shown in black, as well as the polyphyletic CPXVs, which are color coded. The sequence differences compared to VACV/VARV are highlighted in red. Because of their polyphyletic behavior, the CPXVs could be subdivided into seven different variants. Red CPXV I; green CPXV II; blue CPXV III; violet CPXV IV; yellow CPXV V; bright green CPXV VI and CPXV VII.

The highest number of variations was found within the four antigenic sites of the epitope complex #1A–D located at an A27 surface domain between aa 26–39 (KKPEAKREAIVKAD; epitope #1A: aa 32–39, #1B: aa 28–33, #1C: aa 26–31, #1D: 28–34). This complex is highly conserved in sequences of VARV major (66/67), VARV minor (2/2), VACVs (59/61), BPXV (26/26), HSPV (2/2), RPXV (2/2) and TaPXV (2/3) (Tables S4, S5 and S7). All CMLVs (18/18) showed a unique aa exchange V36I leading to the motif 26-KKPEAKREAI**I**KAD-39. The exchanges A30D, R32H, and I35T (26-KKPE**D**K**H**EA**T**VKAD-39) were characteristic for ECTV (14/14). The three aa exchanges K27N, A30T and D39Y were specific for all MPXVs (57/57) and resulted in the motif 26-K**N**PE**T**KREAIVKA**Y**-39. The New World OPXVs, including RCNV (1/1), SkPXV (3/3) and VPXV (1/1) showed the most different, however, species-specifically conserved aa sequence. In CPXVs, a much more polyphyletic arrangement was found, resulting in seven different CPXV motifs (Figure 5). These results were confirmed by the peptide microarrays including various variants (Figure S3 and Table S1).

### 3.7. Silent Mutations in the Epitope Sequences among the OPXV Members

When looking at the nucleotide sequences within the regions coding for the epitopes #1A–D, #4, and #5, we also searched for silent mutations in all three epitope regions. VACV (61/61) and its variants including HSPV (2/2), BPXV (26/26) and RPXV (2/2) showed no silent mutations. However, within the monophyletic groups such as CMLV (n: 17/18; ntx: gac28–30gat), ECTV (n: 12/14; ntx: gac28–30gat and n: 14/14; ntx: ata202–204atc), SkPXV (n: 3/3; ntx: gac28–30gat, gat31–33gac, gca37–39gcg, aag79–81aaa, cgc94–96cgt100–102, gcc112–114gca142–144, gaa283–285gag, aag286–288aaa and tgt289–291tgc) and VARV major and minor (n: 68/69; ntx: gac28–30gat) a few silent mutations were confirmed, and could be used for classification, because they were species-specific. In the case of the polyphyletic CPXVs, we found eight silent mutations in the epitope coding regions of the A27L gene (n: 5/134 ntx: gac28–30gat; n: 19/134 ntx: ctt34–36ctc; n: 42/134 ntx: aaa76–78aag; n: 1/134 ntx: aag79–81aaa; n: 4/134 ntx: cgc94–96cgt; n: 1/134 ntx: gca100–102gcc; n: 85/134 ntx: gcc112–114gct; n: 32/134 ntx: ata202–204atc). Due to their irregular distribution among the different CPXV strains, they were not suitable for a taxonomic classification.

## 4. Discussion

The A27 protein is immunogenic and highly conserved within the members of OPXV [32,61,65]. In this study, six antigenic sites on the A27 protein (epitope #4: aa region 9–14, epitope complex #1A–D: between aa 26 and 39 and epitope #5: aa region 68–71) were mapped. With respect to epitopes #4 and #1A–D, the mapping with the SPOT synthesis and microarray chip showed similar results with only few amino acids divergence. By contrast, epitope #5 could not be detected when the OPXV microarray chip was employed. Although the granularity of membranes is higher with an offset of one, compared to an offset of three in the microarrays, also the resolution of the microarray slide is sufficient to detect epitope #5 with a length of four spots. One reason may be degradation of the respective antibodies over time, as a time lag >5 years between the former spot measurement and the more recent microarrays existed. Another reason may be the use of different side chain protection groups. For production of membranes, Cys(acetyl-aminomethyl) was used, whereas for peptides in the microarray assay Cys(triphenylmethyl) was used that was deprotected during slide preparation via trifluoroacetic acid (TFA). As a cysteine residue is involved in binding, a formation of disulfide bonds or incomplete Cys deprotection may have altered antibody binding properties, thereby leading to different signals in the microarrays compared to the membranes [83]. The OPXV microarray chip, however, was used to screen for additional species-specific epitope variations by aa exchanges according to the GenBank database entries.

Epitope #4 was conserved among all OPXVs and nearly genus-specific, because the main motif 9-DDDLAI-14 was found in 372/391 database entries. In 13 OPXV strains, the N-terminal sequences could not be assessed due to truncations in the sequences uploaded from GenBank. In 3/26 BPXVs, the motif was 9-DDDLAT-14, whereas all three SkPXVs contain the motif 9-DDDMAI-14. The changed aa sequence (L12M) was represented by spot 479 of the OPXV microarray chip and mAb 2G8/1E4 reacted with this spot (Table S7). In 2D predictions of the secondary structure of the A27 protein, a β-turn was evident in this area with a high antigenicity. This was expected as in previously published data epitopes often were identified in the region of β-turns [84,85,86,87]. Predictions on hydrophilicity [88] and surface probability [89] did not show any special features for this region of the A27 protein. Nevertheless, the mAb 2G8/1E4 against epitope #4 showed equally good binding affinities to all OPXV reference strains tested. Its neutralization capacity could be enhanced by the addition of complement. However, in previous studies the mAb 2G8/1E4 showed no neutralizing abilities against the tested ECTV M1 [40]. The discrepancy is caused by another test setup including another OPXV strain (VACV Elstree instead of ECTV M1) and different cells (Vero cells instead of MA-cells) as well as by a 20-fold higher initial concentration of the antibody. The same was true for mAbs 3F5/2D5 and 1D5/2D11 (epitope complex #1 as mentioned further below).

Epitope #5 was the most highly conserved one in A27 as the motif 68-IEKC-71 was present in 389/391 aa sequences. In all three SkPXVs and 1/61 VACV sequences the epitope was shifted downstream to 93-IEKC-96. In 1/134 CPXV strains, the motif was 68-IEKY-71 while in another CPXV the epitope was missing because the C-terminus was truncated. The mAb 5B1/2G6 against epitope #5 was not neutralizing. However, the antigenic site is located in a functionally very important area within the C-terminus of the A27 protein. In this hydrophilic region, the two cysteines at positions 71 and 72 are responsible for formation of disulfide bonds and, therefore, play an important role for a functionally active trimeric A27 structure [52,79]. In 2D predictions of the secondary structure, a β-sheet (aa 58–83) followed by two β-turns (aa 70–75) was evident in this constant area. Two β-turns led generally to a high antigenicity [85]. Predictions on hydrophilicity [88] and surface probability [89] did not reveal any special features. The mAb 5B1/2G6 showed similar binding affinities to all OPXV reference strains tested. This was expected because of the high sequence conservation of the targeted epitope among OPXV.

The most important antigenic region of the A27 protein was confined by aa 26–39. This has already been known from a previous investigation that identified functional domains in the A27 envelope protein [90]. In our present study, a complex of four closely related epitopes (#1A–D) could be allocated to this region. The narrow location of these epitopes has already been predicted previously from data obtained with two overlapping oligopeptides [91] and from quantitative competitive ELISAs performed with purified mAbs and viruses [40]. Two of the four mAbs binding to these epitopes were neutralizing in vitro. In this study, the four mAbs could enhance virus inhibition after adding complement. Other authors identified this region also as a strong target for binding of mAbs [42]. An epitope with a larger extension (aa 21–40) comprised the area of the epitope complex #1A–D completely (aa 26–39). However, those mAbs directed to this epitope region only neutralized in the presence of complement. According to 2-D structure predictions and published data [52,91,92], the A27 region with the four epitopes #1A–D was classified as hydrophilic. Between aa residues 25 and 45 a hypervariable structure region was found. In case of VACV and VARV, it started with an α-helix up to aa 40, followed by two β-turns. In CPXV and CMLV, however, the α-helix changed at aa 37 into three and four β-turns, respectively. MPXV showed two β-turns at aa residues 25–34 followed by an α-helix up to aa 39 and three β-turns. In ECTV, the β-sheet structure was found up to aa 30, followed by an α-helix up to aa 37 and three β-turns. These highly variable structural conditions led to a significant species-specific difference in the overall structure of the investigated A27 proteins. Thus, the proteins of the species VACV, VARV and CMLV had a more linear form, while the proteins of the species MPXV, CPXV and ECTV were folded to a larger extent. Considering the epitope complex #1A–D, the aa main motif was 26-KKPEAKREAIVKAD-39. Based on GenBank database entries for 391 A27 protein sequences, this motif in the complete form (aa 26–39) was found in 210/391 OPXVs (68/69 VARV, 59/61 VACV, 26/26 BPXV, 2/2 HSPV, 2/2 RPXV, 51/134 CPXV, 2/3 TaPXV). The database entries for the 391 A27 protein sequences also indicated that this region could be defined as a very variable area with a lot of aa exchanges and structural differences. Affinity experiments showed, that the binding of the four mAbs to their respective targets was different and obviously dependent on aa exchanges. The epitopes #1A and #1B were completely absent in MPXV and ECTV. Especially in case of MPXV, three aa exchanges led to the motif variation 26-K**N**PE**T**KREAIVKA**Y**-39, independent of the geographic distribution of isolates. In ECTVs, three different aa exchanges in comparison to VACV led to the motif to 26-KKPE**D**K**H**EA**T**VKAD-39. In both OPXV genera, these aa exchanges were absolutely species-specific (57/57 MPXV; 14/14 ECTV). To investigate the direct influence of the exchanged amino acids on the binding of the corresponding mAbs, the epitope #1A was re-synthesized on a SPOTs membrane in the unchanged (VACV) and changed (MPXV and ECTV) design. The aa exchanges led unequivocally to the loss of mAb binding to its epitope #1A (Figure S4) which was confirmed by lack of binding to spots 488 and 489 on the OPXV microarray chip (Table S7). The variations between aa 26 and 39 led also to a significant structural change of the A27 protein homologs of MPXV and ECTV. The change of the aspartic acid at position 39 of MPXV to tyrosine, containing a benzene ring, was mainly responsible for the loss of the mAb reactivity. The binding site of the main immunogenic epitope #1A was defined as an octapeptide of 31-REAIVKAD-39, when the three decapeptides on the SPOTs membrane with the highest spot intensity (No. 11–13) were taken for epitope determination (Figure S2). However, in all seven decapeptides (No. 10–16), even those with weaker reactions, used for the evaluation, it became clear that the tetrapeptide 35-IVKA-38 was the most important factor for binding of the mAb 5B4/2F2 (epitope 1A). It was apparent, that the VACV tetrapeptide 35-IVKA-38 was present in the CMLV variation 35-IIKA-38. If the CMLV specificity is referred to the whole region of epitope complex #1A-D, database analysis will reveal that the aa exchange V36I is unique for all 18/18 CMLVs, independent of their geographical origin (Africa, Asia), thereby leading to the two motifs 26-KKPEAKREAI**I**KAD-39 (17/18) and 26-K**R**PEAKREAI**I**KAD-39 (1/18).

The most prominent but species-specifically conserved aa sequence difference in the epitope complex #1A–D was found in New World OPXVs with the motifs 26-**_**KPEAKR**KVVE**KAD-39 in RCNV (1/1), 26-**_**KPE**E**KR**K**A**V**VKA**E**-39 in VPXV (1/1), and 26-KKPE**E*PV***KR**KVV*KNKNKHKV*V**KAD-49 in SkPXV (3/3). In spite of the 4 aa exchanges in RCNV, the mAbs against epitopes #1B–D gave a weak signal on spot 490 of the OPXV microarray chip (Table S7), whereas the epitope #1A was not detected. In VPXV and SkPXV (spots 491 and 492), the epitope complex #1A–D could not be detected by any of the mAbs.

Because of the fact that the sequence differences in the A27 region, representing the epitope complex #1A–D, were species-specifically conserved, the Old World OPXVs, such as VARV, VACV, HSPV, RPXV, BPXV, ECTV, MPXV, CMLV, and TaPXV, as well as the New World OPXVs, like SkPXV, RCNV and VPXV, revealed a monophyletic character. The sequence variations in this area, however, were not species-specifically conserved in CPXVs, which is why this group was regarded as polyphyletic. This taxonomic arrangement was concordant with previous investigations, where CPXVs were classified into different clades based on whole genome analysis [93,94,95]. According to the most recent findings, CPXVs were divided into four clades, CPXV-like 1, CPXV-like 2, VACV-like and VARV-like [93]. In our study, we could identify seven CPXV variants when referring only to the amino acid sequences of the epitope complex #1A–D (Figure 5).

The A27 protein was formerly categorized as a fusion protein [32,52,54] and believed to mediate the direct fusion of virus and cytoplasm membranes (“fusion from without”) [52,79]. Hitherto, A27 is not settled to be a part of a fusion complex consisting of at least 11 different proteins (A16, A21, A28, F9, G3, G9, H2, J5, L1, L5 and O3), being conserved in all OPXVs [36,37,81]. Still, there is also evidence in the literature that the A27 and A17 proteins form a second fusion complex [65], which was assigned to fusion proteins type I. Typical for type I viral fusion proteins is the presence of a coiled-coil structure [59], which is, beside the A27, also seen in influenza virus HA2 [96], Ebola GP2 [97,98] and HIV gp41 [99]. The authors suggested that the A17-A27-complex is transported to the cell membrane during viral replication and mediates fusion of the infected cells (“fusion from within”), meaning that A17 is the membrane-anchoring domain with the fusion peptide (aa 18–34) and A27 is responsible for the oligomerization as well as the membrane-attachment [65]. A27 binds to the GAG heparan sulfate of neighboring cells. This binding is mediated through the aa residues 26-KKPE-29 [34,57,61,62], resulting in an accumulation of cells in the immediate vicinity [65]. In several studies, mAbs against the A27 protein were able to block the “fusion from within” in a model described previously [32,52,54]. Therefore, we used this model to test inhibition of the fusion by three anti-A27-mAbs from our collection, whose antigenic sites were exactly mapped. Through low-pH treatment [54,100], we were able to induce fusion of VACV infected cells. This “fusion from within” was indicated by the formation of large and structureless fused cell areas known as syncytia [54]. The mAb 3F5/2D5 directed to epitope #1C (aa 26-KKPEAK-31) was not able to block the fusion, although the GAG binding site being inside its epitope. Acid-induced syncytia were formed. By adding the mAb 5B4/2F2 directed to epitope #1A (aa 32-REAIVKAD-39) and binding just more to the C-terminus of the mAb 3F5/2D5, fusion could be inhibited. The reason for the inhibition is not clear at the moment. However, at least a steric hindrance of the mAb could be ascertained. Moreover, antibodies may directly interfere with interactions by occupying binding sites or sterically hindering binding sites in close proximity. In addition, antibody binding affects protein conformation, and different antibodies have different effects on protein conformation that may alter distant interacting sites. The mAb 5B1/2G6 binding to the C-terminal epitope #5 (aa 68–71) failed to block the fusion by showing polykaryon formation, too. The epitope of this mAb is directly related to the binding site (aa 71–72) of the A26 fusion suppressor protein to the A27 protein, but there was no direct influence on the fusion event.

In summary, we mapped six antigenic sites on the A27 protein of VACV. This enabled us to interpret species-specific epitope variations and conservations of various OPXVs to gain an impression of their phylogenic relationships. To elucidate structure function relationships in more detail, co-crystallization might be helpful for future investigations. Moreover, the data on antigenic sites for cross-reacting or monospecific neutralizing antibodies are of high relevance for target directed screening of human immunoglobulin libraries to generate specifically engineered human recombinant antibodies, which might help in controlling any future outbreak of zoonotic orthopoxviruses.

## Figures and Tables

**Table 1:** Neutralization efficiency of six different purified anti-A27 monoclonal antibodies against several epitopes with and without complement binding.

Epitope	Position (aa)	MAb	Virus Strain Used for mAb Production	Isotype	Neutralization without Complement (µg/mL)	Neutralization with 1% Complement (µg/mL)
1A	32–39	5B4/2F2	VACV MVA	IgG2a	12.5	1.6
1B	28–33	2C11/1B4	VACV MVA	IgG2b	25.0	3.1
1C	26–31	3F5/2D5	CPXV KR2 Brighton	IgG1	-	200.0
1D	28–34	1D5/2D11	CPXV KR2 Brighton	IgG1	-	100.0
4	9–14	2G8/1E4	ECTV Munich 1	IgG3	200.0	12.5
5	68–71	5B1/2G6	ECTV Munich 1	IgG2a	-	-

-: No neutralization observed.

## Supplementary Materials

The following are available online at https://www.mdpi.com/1999-4915/11/6/493/s1: Figure S1: Layout of the poxvirus microarray chip. A: The chip contains eight arrays arranged in the same manner. B: Arrangement of spots on each array: yellow is peptides representing A27 antigen of Vaccinia virus Western Reserve; red, D8; blue, H3; grey, L1; green, A33; orange, B5. The lower part of the array contains peptide sequence variations of A27 (lilac) and D8 (pink) antigens of the other orthopoxviruses. Sites of the sample deposition depicted by the plus symbol. The number serves as the batch identification and letters represent the chip name. C: Amino acid sequence of the first three peptides covering A27, corresponding to spots #1–#3 in B. Figure S2: The 14 kDa A27 protein of VACV Copenhagen was synthesized on a SPOTs membrane as 101 decapeptides with 9 aa overlap to cover the whole sequence of 110 aa. By immunodetection with the mAb 5B4/2F2 seven spots were identified (No. 10–16) to carry the target sequences spots 11–13 showed the strongest signals. The eight amino acids the three peptides had in common were represented to the sequence region 32-REAIVKAD-39. The minimal sequence essential for binding is 35-IVKA-38 and marked in red. Figure S3: The OPXV microarray is based on 521 15-mer peptides overlapping by 12 aa. These peptides were spotted by SPOT technique on a chip. After immunodetection, the responding spots with the highest common intensity were chosen and outlined. Epitope #1A (mAb 5B4/2F2) was directed to a sequence region aa 31-KREAIVKAD-39. Epitopes #1B (mAb 2C11/1B4), #1C (mAb 3F5/2D5) and #1D (mAb 1D5/2D11) were all assigned to the aa region 28-PEAKRE-33. Epitope #4 (mAb 2G8/1E4) was allocated to aa 7-PGDDDLAIPATE-18. MAb 5B1/1A11 (epitope #5), however, did not react with any of the peptides on the chip. Figure S4: Immunological detection of ECTV- and MPXV-specific aa exchanges of the epitope #1A sequence on SPOTs membrane by the mAb 5B4/2F2. Spot 89: VACV epitope #1A (32-REAIVKAD-39). Spot 90: MPXV epitope #1A (32-REAIVKA**Y**-39). Spot 91: ECTV epitope #1A (32-**H**EA**T**VKAD-39). The aa exchanges led unequivocally to the loss of the binding of the mAb 5B4/2F2. The weak response on spot 91 was also observed with the secondary antibody used for detection. Table S1: Reactivity of six A27-specific mAbs on the OPXV microarray chip. The amino acids marked in red are species-specific sequence variations to the VACV WR or epitope sequence exchanges. *Accession number is representative for several sequences of this type. All GenBank accession numbers can be found in Table S4. Different shades of grey represent strength of the fluorescence intensity. n.d.: not detected

Table S2: Detection of the six A27 antigenic sites by the corresponding anti-OPXV-mAbs via SPOTs-membrane and OPXV peptide microarray chip. The matches within the epitope sequence are highlighted and underlined. Table S3: Mapping of epitope #4 based on 391 complete and partial amino acid sequences from the NCBI GenBank database. Differences within the epitope sequence are highlighted. Table S4: 391 amino acid sequences of the OPXV A27 protein homologs available so far in the GenBank, being analyzed with respect to species-specific conservation or variation of the six sequential antigenic sites mapped. Table S5: Nucleotide sequences of the OPXV A27 protein homologs available so far in the GenBank, showing mutations which are crucial for amino acid exchanges as well as silent mutations. Table S6: Mapping of epitope #5 based on 391 complete and partial amino acid sequences from the NCBI GenBank database. Differences within the epitope sequence are highlighted. Table S7: Mapping of epitope complex #1A–D based on 391 complete and partial amino acid sequences from the NCBI GenBank database. Differences within the epitope sequence are highlighted.
