# Supplementary material for: Species-Specific Conservation of Linear Antigenic Sites on Vaccinia Virus A27 Protein Homologs of Orthopoxviruses
Source: Viruses. 2019 May 29;11(6):493. doi: 10.3390/v11060493 (PMC6631127; doi:10.3390/v11060493)
Supplement: Supplementary file 1 [file viruses-11-00493-s001.zip › AhsendorfH2019_FigS2.pdf]

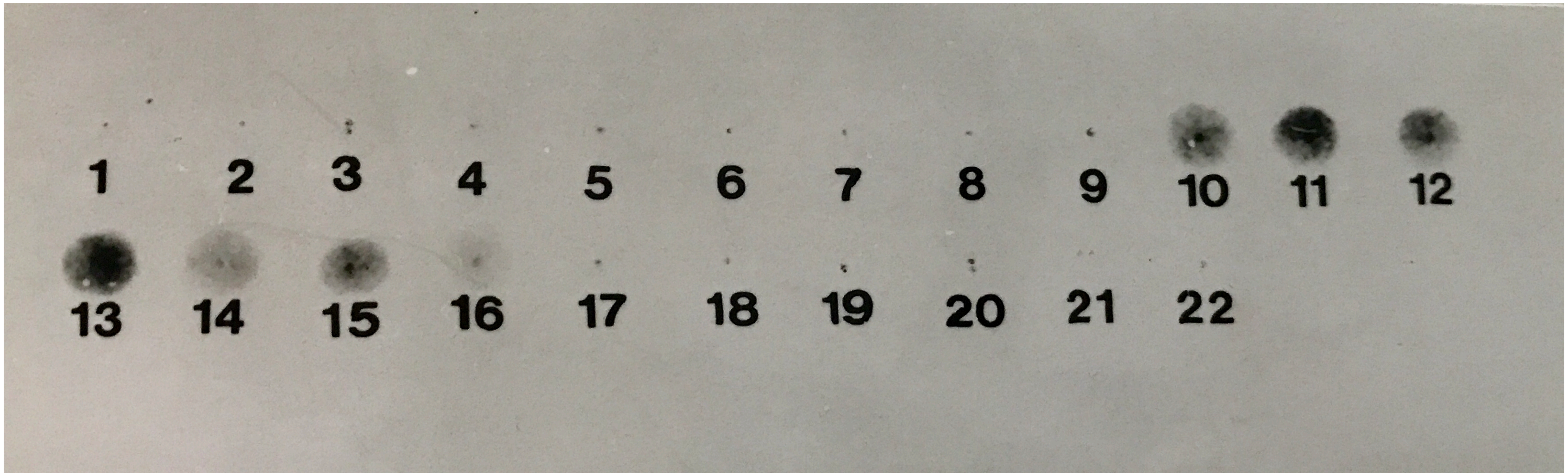

Peptide	Sequence
Peptide Nr. 10:	E A K R E A **I V K A**
Peptide Nr. 11:	A K **R E A I V K A D**
Peptide Nr. 12:	K **R E A I V K A D** E
Peptide Nr. 13:	**R E A I V K A D** E D
Peptide Nr. 14:	E A **I V K A** D E D D
Peptide Nr. 15:	A **I V K A** D E D D N
Peptide Nr. 16:	**I V K A** D E D D N E
