# Supplementary material for: Species-Specific Conservation of Linear Antigenic Sites on Vaccinia Virus A27 Protein Homologs of Orthopoxviruses
Source: Viruses. 2019 May 29;11(6):493. doi: 10.3390/v11060493 (PMC6631127; doi:10.3390/v11060493)
Supplement: Supplementary file 1 [file viruses-11-00493-s001.zip › AhsendorfH2019_FigS4.pdf]

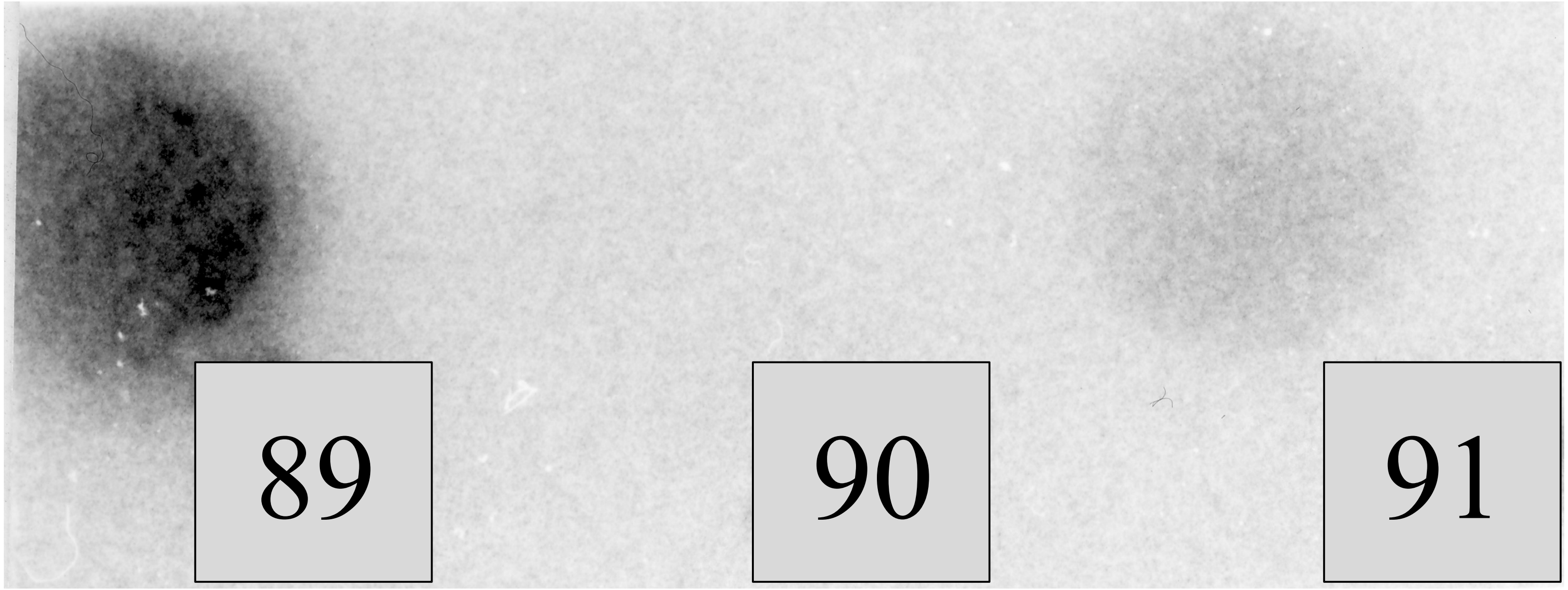

Spot 89: Epitope 1A in VACV Copenhagen: REAIVKAD

Spot 90: Epitope 1A in MPXV Copenhagen: REAIVKA**Y**

Spot 91: Epitope 1A in ECTV Munich 1: **H**EA**T**VKAD

aa 32 33 34 35 36 37 38 39
