# Supplementary material for: Species-Specific Conservation of Linear Antigenic Sites on Vaccinia Virus A27 Protein Homologs of Orthopoxviruses
Source: Viruses. 2019 May 29;11(6):493. doi: 10.3390/v11060493 (PMC6631127; doi:10.3390/v11060493)
Supplement: Supplementary file 1 [file viruses-11-00493-s001.zip › AhsendorfH2019_supp_table2.pdf]

Table S2 Detection of the six A27 antigenic sites by the corresponding anti-OPXV-mAbs *via* SPOTs-membrane and OPXV peptide microarray chip.

Epitope	Epitope sequences determined on SPOTs-membrane:		Epitope sequence determined on OPXV peptide microarray:	
	**AA position**	**AA composition**	**AA position**	**AA composition**
1A	32-39	**REAIVKAD**	31-39	K**REAIVKAD**
1B	28-33	**PEAKRE**	28-33	**PEAKRE**
1C	26-31	KK**PEAK**	28-33	**PEAK**RE
1D	28-34	**PEAKRE**A	28-33	**PEAKRE**
4	9-14	**DDDLAI**	7-18	PG**DDDLAI**PATE
5	68-71	IEKC	-	Not detected

The matches within the epitope sequence are highlighted and underlined.
