# Supplementary material for: Species-Specific Conservation of Linear Antigenic Sites on Vaccinia Virus A27 Protein Homologs of Orthopoxviruses
Source: Viruses. 2019 May 29;11(6):493. doi: 10.3390/v11060493 (PMC6631127; doi:10.3390/v11060493)
Supplement: Supplementary file 1 [file viruses-11-00493-s001.zip › AhsendorfH2019_supp_table3.pdf]

Table S3 Mapping of epitope #4 based on 391 complete and partial amino acid sequences from the NCBI GenBank database.

**Linear A27 epitope aa 9-14**	**OPXV genera**	**Number of DB entries**
DDDLAI	VARV major	66/67
	VARV minor	2/2
	VACV	60/61
	BPXV	23/26
	HSPV	2/2
	RPXV	2/2
	CMLV	17/18
	CPXV	128/134
	ECTV	13/14
	MPXV	57/57
	TaPXV	2/3
DDDLA**T**	BPXV	3/26
DDD**M**AI	SkPXV	3/3
**N-Terminus truncated**	VARV major	1/67
	CMLV	1/18
	CPXV	6/133
	ECTV	1/14
	TaPXV	1/3
	RCNV	1/1
	VPXV	1/1
**RARSPR**	VACV	1/61

Differences within the epitope sequence are highlighted.
