# Supplementary material for: Species-Specific Conservation of Linear Antigenic Sites on Vaccinia Virus A27 Protein Homologs of Orthopoxviruses
Source: Viruses. 2019 May 29;11(6):493. doi: 10.3390/v11060493 (PMC6631127; doi:10.3390/v11060493)
Supplement: Supplementary file 1 [file viruses-11-00493-s001.zip › AhsendorfH2019_supp_table1.pdf]

Table S1 Reactivity of six A27-specific mAbs on the OPXV microarray chip. The amino acids marked in red are species-specific sequence variations to the VACV WR or epitope sequence exchanges.

Spot ID	Spot Designation	Sequence Variations	Epitope 1A	Epitope 1B	Epitope 1C	Epitope 1D	Epitope 4	Epitope 5
			Epitope sequence according to SPOTs Membrane					
			REAIVKAD	PEAKRE	KKPEAK	PEAKREA	DDDLAI	IEKC
			Epitope sequence according to OPXV Microarray Chip					
			KREAIVKAD	PEAKRE	PEAKRE	PEAKRE	PGDDDLAIPATE	n.d.
2	VACV WR (AAO89429.1)	TLFPGDDDLAIPATE						
3		PGDDDLAIPATEFFS						
478	VACV WR (AAO89429.1)	LFPGDDDLAIPATEF						
479	SkPXV (AAQ72922.1)*	LFPGDDDMAIPATEF						
7	VACV WR (AAO89429.1)	FFSTKAAKKPEAKRE						
8		TKAAKKPEAKREAIV						
9		AKKPEAKREAIVKAD						
10		PEAKREAIVKADEDD						
11		KREAIVKADEDDNEE						
481	VARV (ABF26518.1)	AKKPEAKHEAIVKAD						
482	VACV (AAA48152.1)*	DKKPEAKREAIVKAD						
522	CMLV (CAA52999.1)*	AKKPEAKREAIIKAD						
483	CPXV (AAP48887.1)*	AKKPEAKREAIVKAE						
484	CPXV (AAP48888.1)	AKKPEAKREAFVKAE						
485	CPXV (AAQ72894.1)*	AKKQEAKREAIVKAE						
486	CPXV (AAQ72906.1)	AKKPEAKHEAIVKAE						
488	ECTV (CAA53000.1)*	AKKPEDKHEATVKAD						
489	MPXV (CAA52998.1)*	AKNPETKREAIVKAY						
490	RCNV (ABD37609.1)	AKKPEAKRKVVEKAD						
491	VPXV (ABD37608.1)	AKKPEEKRKAVVKAE						
492	SkPXV (AAQ72922.1)*	AKKPEEPVKRKVVKN						
497	VARV (CAA47501.1)*	TKFEQIEKCCKRNDE						

*Accession number is representative for several sequences of this type. All GenBank accession numbers can be found in Table S2. Different shades of grey represent strength of the fluorescence intensity.

n.d.: not detected

Weak reaction

Strong reaction
