# Supplementary material for: Species-Specific Conservation of Linear Antigenic Sites on Vaccinia Virus A27 Protein Homologs of Orthopoxviruses
Source: Viruses. 2019 May 29;11(6):493. doi: 10.3390/v11060493 (PMC6631127; doi:10.3390/v11060493)
Supplement: Supplementary file 1 [file viruses-11-00493-s001.zip › AhsendorfH2019_supp_table7.pdf]

Table S7 Mapping of epitope complex #1A-D based on 391 complete and partial amino acid sequences from the NCBI GenBank database.

Linear A27 epitope aa 26-39	OPXV genera	Number of DB entries
KKPEAKREAIVKAD	VARV major	66/67
	VARV minor	2/2
	VACV	59/61
	BPXV	26/26
	HSPV	2/2
	RPXV	2/2
	CPXV	51/134
	TaPXV	2/3
_KPEAKREAIVKAD	CPXV	3/134
	TaPXV	1/3
KKPEAKREAIVKA**E**	CPXV	64/134
_KPEAKREAIVKA**E**	CPXV	2/134
KKPEAKREAI**I**KAD	CMLV	17/18
K**R**PEAKREAI**I**KAD	CMLV	1/18
K**R**PEAKREAIVKA**E**	CPXV	1/134
K**N**PE**T**KREAIVKA**Y**	MPXV	57/57
KKPE**D**K**H**EA**T**VKAD	ECTV	13/14
_KPE**D**K**H**EA**T**VKAD	ECTV	1/14
KKPEAK**H**EAIVKAD	VARV major	1/67
KKPEAK**H**EAIVKA**E**	CPXV	1/134
KK**Q**EAKREAIVKA**E**	CPXV	8/134
_K**Q**EAKREAIVKA**E**	CPXV	1/134
KKPEAKREA**F**VKA**E**	CPXV	1/134
KKPE**V**KREAIVKA**E**	CPXV	1/134
_KPE**E**KR**K**A**V**VKA**E**	VPXV	1/1
KKP**DR**KRE**Q**IVKAD	VACV	1/61
_KPEAKR**KVVE**KAD	RCNV	1/1

K**SQRLNAKQLLKLR**	CPXV	1/1
**TSRSSTGS**A**NPS**A**S**	VACV	1/61
KKPE**EPV**KR**KVVKNKNKHKV**VKAD **(aa 26-49)**	SkPXV	3/3

Differences within the epitope sequence are highlighted.
