# Supplementary material for: Species-Specific Conservation of Linear Antigenic Sites on Vaccinia Virus A27 Protein Homologs of Orthopoxviruses
Source: Viruses. 2019 May 29;11(6):493. doi: 10.3390/v11060493 (PMC6631127; doi:10.3390/v11060493)
Supplement: Supplementary file 1 [file viruses-11-00493-s001.zip › AhsendorfH2019_supp_table6.pdf]

Table S6 Mapping of epitope #5 based on 391 complete and partial amino acid sequences from the NCBI GenBank database.

Linear A27 epitope aa 68-71	OPXV genera	Number of DB entries
IEKC	VARV major	67/67
	VARV minor	2/2
	VACV	60/61
	BPXV	26/26
	HSPV	2/2
	RPXV	2/2
	CMLV	18/18
	CPXV	132/134
	ECTV	14/14
	MPXV	57/57
	TaPXV	3/3
	RCNV	1/1
	VPXV	1/1
**(aa 93-96)**	SkPXV	3/3
**(aa 93-96)**	VACV	1/61
IEK**Y**	CPXV	1/134
**C-Terminus truncated**	CPXV	1/134

Differences within the epitope sequence are highlighted.
