# Supplementary figures and images for: Species-Specific Conservation of Linear Antigenic Sites on Vaccinia Virus A27 Protein Homologs of Orthopoxviruses
Source: Viruses. 2019 May 29;11(6):493. doi: 10.3390/v11060493 (PMC6631127; doi:10.3390/v11060493)

A

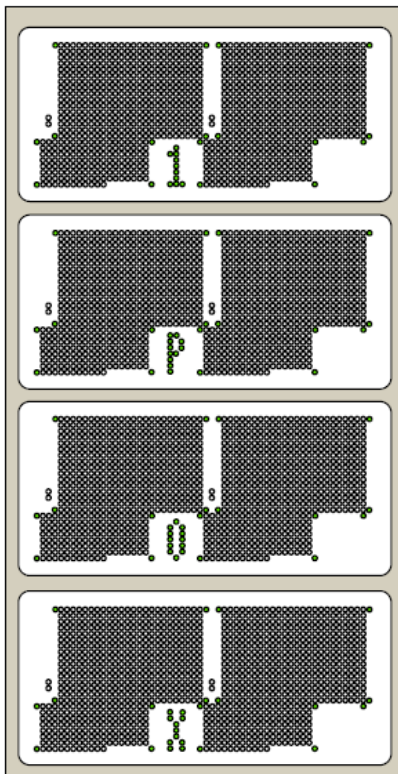

B

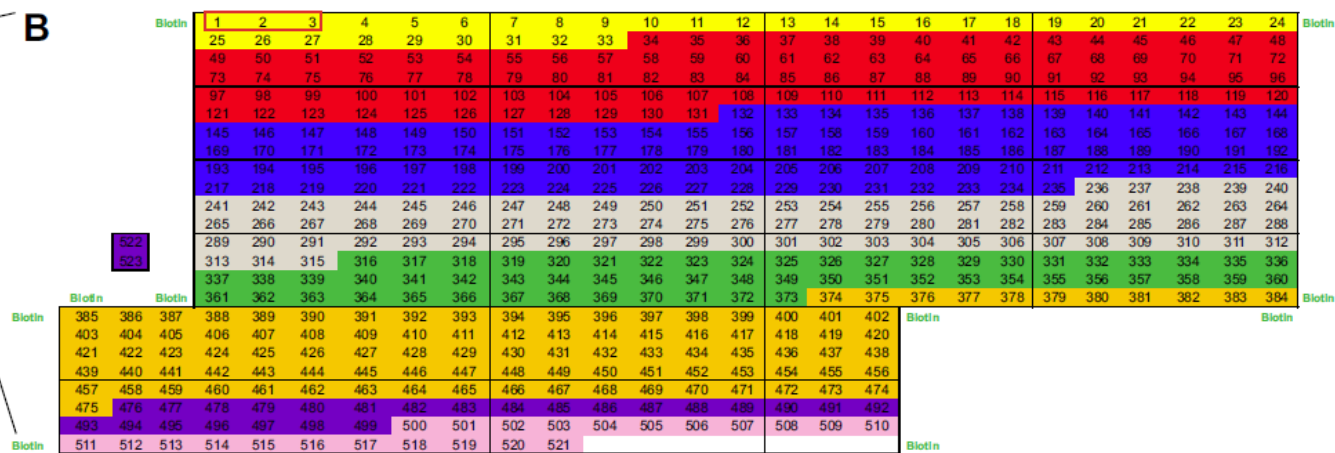

C

| Spot # | Amino acid sequence |
|--------|---------------------|
| 1      | MDGTLFPGDDDLAIP     |
| 2      | TLFPGDDDLAIPATE     |
| 3      | PGDDDLAIPATEFFS     |

Supplement: Supplementary file 1 [file viruses-11-00493-s001.zip › AhsendorfH2019_FigS1.pdf]

**Epitope 1A (MAb 5B4/2F2)**

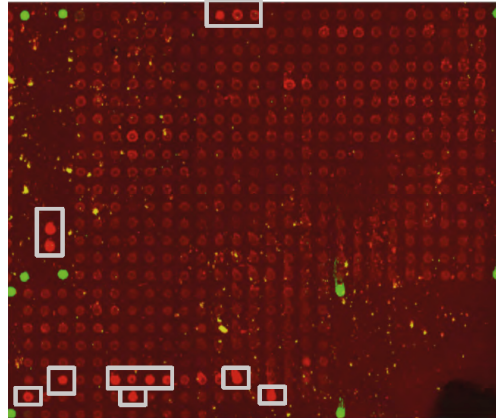

**Epitope 1B (MAb 2C11/1B4)**

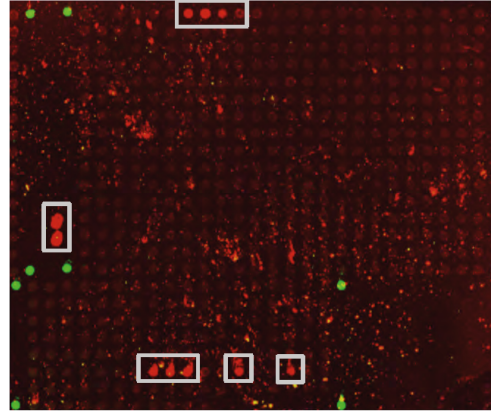

**Epitope 1C (MAb 3F5/2D5)**

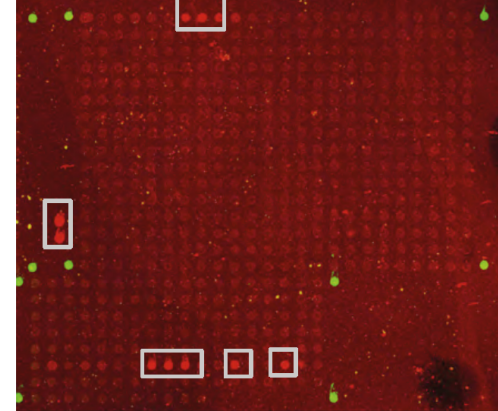

**Epitope 1D (MAb 1D5/2D11)**

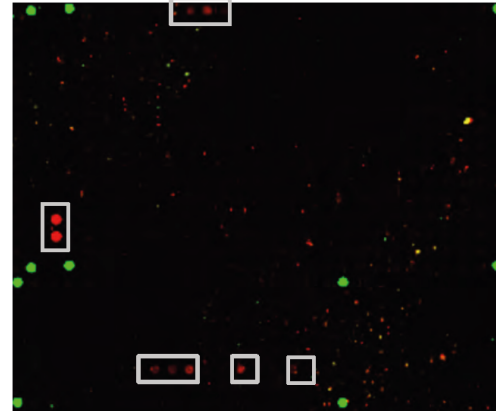

**Epitope 4 (MAb 2G8/1E4)**

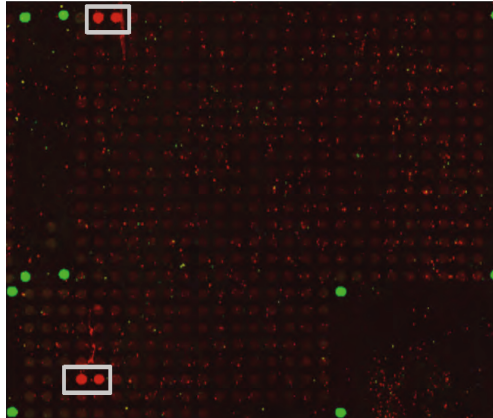

**Epitope 5 (MAb 5B1/2G6)**

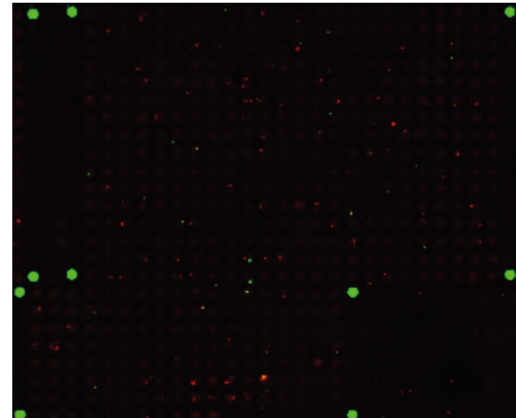

Supplement: Supplementary file 1 [file viruses-11-00493-s001.zip › AhsendorfH2019_FigS3.pdf]
